# 2075. Multidrug-Resistant Organism (MDRO) Co-Carriage Patterns in Nursing Homes (NHs): Methicillin-resistant *Staphylococcus aureus* (MRSA), Vancomycin-resistant *Enterococci* (VRE), Extended Spectrum beta-lactamase (ESBL) producers, Carbapenem-resistant Enterobacterales (CRE), Carbapenem-resistant *Acinetobacter baumannii* (CRAB), and *Candida auris*

**DOI:** 10.1093/ofid/ofad500.145

**Published:** 2023-11-27

**Authors:** Gabrielle Gussin, Raveena D Singh, Raheeb Saavedra, Connie Nguyen, John Mitchell, Kristine Nguyen, Gabriel Gadia, Steven Vu, Alice Lee, Julie A Shimabukuro, Cassiana E Bittencourt, Susan S Huang

**Affiliations:** University of California, Irvine School of Medicine, Division of Infectious Diseases, Irvine, California; University of California, Irvine School of Medicine, Division of Infectious Diseases, Irvine, California; University of California, Irvine School of Medicine, Division of Infectious Diseases, Irvine, California; University of California, Irvine, Irvine, California; University of California, Irvine, Irvine, California; University of California, Irvine, Irvine, California; University of California, Irvine, Irvine, California; University of California, Irvine, Irvine, California; University of California, Irvine, Irvine, California; University of California, Irvine Health, Orange, California; University of California, Irvine Health, Orange, California; University of California, Irvine School of Medicine, Irvine, CA

## Abstract

**Background:**

NHs are high-risk settings for MDRO spread.

**Methods:**

We evaluated NH MDRO prevalence and co-carriage patterns in 22 NHs in Orange County, CA from Fall 2022-Spring 2023. 25 MDRO sweeps each involved 50 randomly-sampled occupied beds. Residents had swabs collected from bilateral axilla/groin and peri-rectal areas (MRSA, VRE, ESBL, CRE, CRAB, *C. auris*), and from bilateral nares (MRSA, *C. auris*). We assessed overall MDRO prevalence, as well as MDRO co-carriage patterns across organisms and MDRO body site carriage for each pathogen.

**Results:**

Of 1250 residents, 57.5% (719) had MDRO carriage at any body site. Prevalence was highest for MRSA (36.7%), followed by ESBL (24.9%), VRE (14.2%), *C. auris* (7.1%), CRAB (2.1%), and CRE (1.0%). Of the 719 MDRO carriers, 62.2% (447) carried 1 MDRO and 37.8% (272) carried ≥2 MDROs. Carriers of one MDRO were likely to carry another (Table 1). For example, MRSA carriers had 48.1% likelihood of carrying another MDRO. Conversely, carriers of any other MDRO had a 45.9% (range: 44.9%-69.2%) likelihood of carrying MRSA. Notably, all CRE carriers and 92.3% of CRAB carriers carried another MDRO. Table 2 shows common body sites for carriage of each MDRO. Nares was the most common site of MRSA carriage; axilla/groin, for CRE and *C. auris*; peri-rectal areas, for VRE and ESBL; and axilla/groin and peri-rectal carriage, for CRAB.

**Figure:**
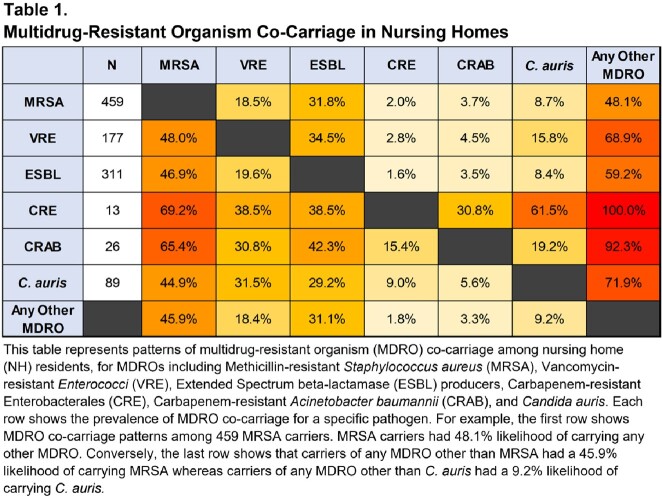

This table represents patterns of multidrug-resistant organism (MDRO) co-carriage among nursing home (NH) residents, for MDROs including Methicillin-resistant Staphylococcus aureus (MRSA), Vancomycin-resistant Enterococci (VRE), Extended Spectrum beta-lactamase (ESBL) producers, Carbapenem-resistant Enterobacterales (CRE), Carbapenem-resistant Acinetobacter baumannii (CRAB), and Candida auris. Each row shows the prevalence of MDRO co-carriage for a specific pathogen. For example, the first row shows MDRO co-carriage patterns among 459 MRSA carriers. MRSA carriers had 48.1% likelihood of carrying any other MDRO. Conversely, the last row shows that carriers of any MDRO other than MRSA had a 45.9% likelihood of carrying MRSA whereas carriers of any MDRO other than C. auris had a 9.2% likelihood of carrying C. auris.

**Figure:**
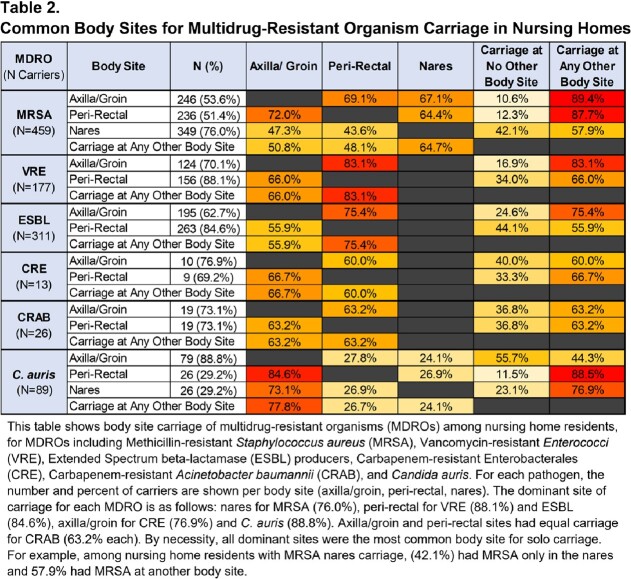

This table shows body site carriage of multidrug-resistant organisms (MDROs) among nursing home residents, for MDROs including Methicillin-resistant Staphylococcus aureus (MRSA), Vancomycin-resistant Enterococci (VRE), Extended Spectrum beta-lactamase (ESBL) producers, Carbapenem-resistant Enterobacterales (CRE), Carbapenem-resistant Acinetobacter baumannii (CRAB), and Candida auris. For each pathogen, the number and percent of carriers are shown per body site (axilla/groin, peri-rectal, nares). The dominant site of carriage for each MDRO is as follows: nares for MRSA (76.0%), peri-rectal for VRE (88.1%) and ESBL (84.6%), axilla/groin for CRE (76.9%) and C. auris (88.8%). Axilla/groin and peri-rectal sites had equal carriage for CRAB (63.2% each). By necessity, all dominant sites were the most common body site for solo carriage. For example, among nursing home residents with MRSA nares carriage, (42.1%) had MRSA only in the nares and 57.9% had MRSA at another body site.

**Conclusion:**

Multi-MDRO carriage is common among NH residents with MDROs harbored at multiple body sites. Co-carriage was especially high among those harboring a carbapenem-resistant organism, likely reflecting accrued MDROs from extensive and repeated antibiotic exposure. Understanding MDRO co-carriage patterns can identify strategies that may be effective across MDROs. For example, nearly half of residents colonized with a non-MRSA MDRO also carried MRSA, suggesting that nasal decolonization of any MDRO carrier may benefit outcomes for MRSA, the most common MDRO in NHs.

**Disclosures:**

**Gabrielle Gussin, MS**, Medline Industries, Inc: Conducted studies where participating hospitals/nursing homes received cleaning & antiseptic product|Xttrium Laboratories: Conducted studies where participating hospitals & nursing homes received antiseptic bathing product **Raveena D. Singh, MA**, Medline Industries, Inc: Conducted studies where participating hospitals/nursing homes received cleaning & antiseptic product|Xttrium Laboratories: Conducted studies where participating hospitals & nursing homes received antiseptic bathing product **Raheeb Saavedra, AS**, Medline Industries, Inc: Conducted studies where participating hospitals/nursing homes received cleaning & antiseptic product|Xttrium Laboratories: Conducted studies where participating hospitals & nursing homes received antiseptic bathing product **Connie Nguyen, n/a**, Xttrium Laboratories: Conducted studies where participating hospitals & nursing homes received antiseptic bathing product **Alice Lee, n/a**, Xttrium Laboratories: Conducted studies where participating hospitals & nursing homes received antiseptic bathing product **Susan S. Huang, MD, MPH**, Medline: Conducted studies in which participating nursing homes received contributed antiseptic bathing and cleaning products|Xttrium: Conducted studies in which participating nursing homes and hospital patients received contributed antiseptic soap

